# Improvement in Low-Homology Template-Based Modeling by Employing a Model Evaluation Method with Focus on Topology

**DOI:** 10.1371/journal.pone.0089935

**Published:** 2014-02-26

**Authors:** Wentao Dai, Tingrui Song, Xuan Wang, Xiaoyang Jin, Lizong Deng, Aiping Wu, Taijiao Jiang

**Affiliations:** 1 Key Laboratory of Protein & Peptide Pharmaceuticals, National Laboratory of Biomacromolecules, Institute of Biophysics, Chinese Academy of Sciences, Beijing, China; 2 University of the Chinese Academy of Sciences, Beijing, China; University of Michigan, United States of America

## Abstract

Many template-based modeling (TBM) methods have been developed over the recent years that allow for protein structure prediction and for the study of structure-function relationships for proteins. One major problem all TBM algorithms face, however, is their unsatisfactory performance when proteins under consideration are low-homology. To improve the performance of TBM methods for such targets, a novel model evaluation method was developed here, and named MEFTop. Our novel method focuses on evaluating the topology by using two novel groups of features. These novel features included secondary structure element (SSE) contact information and 3-dimensional topology information. By combining MEFTop algorithm with FR-t5, a threading program developed by our group, we found that this modified TBM program, which was named FR-t5-M, exhibited significant improvements in predictive abilities for low-homology protein targets. We further showed that the MEFTop could be a generalized method to improve threading programs for low-homology protein targets. The softwares (FR-t5-M and MEFTop) are available to non-commercial users at our website: http://jianglab.ibp.ac.cn/lims/FRt5M/FRt5M.html.

## Introduction

Template-based modeling is defined as modeling of protein structures based on already determined structure templates, and it is currently the most powerful prediction method. To build a structure model for a target sequence, the TBM method usually follows four steps: identification of structural templates, alignment of the target sequence to structural templates (or sequence-structure alignment), model building, and model quality evaluation. In recent years, various TBM programs were developed for the first two steps [1], [2], [3], [4], [5], [6], [7], [8], [9], [10]. In addition, powerful model building tools were developed, including MODELLER and SWISS-MODEL [11], [12]. Lastly, a wide range of tools was developed for the last step, the model quality evaluation [13], [14], [15], [16], [17], [18], [19], [20], [21], [22], [23], [24].

Whilst TBM methods are now widely used for protein structure prediction and structure-function relationship studies, their low performance for low-homology proteins still presents a bottleneck. The underlying reasons behind the bottleneck can be complicated, and include issues like incorrect template selection and sequence-template alignment, modeling errors, or a biased scoring function, to name a few. All together, these errors ultimately result in the failure of generating high-quality models, even in the presence of good templates in the template library at use.

Our previously developed TBM method FR-t5 [7], which has comparable performance to the state-of-the-art fold recognition methods, faces the same problem. In FR-t5, the targets in the dawn region (defined as proteins that have an optimal Z-score <6.0), the ranked 1^st^ models in FR-t5 are always of native-unlike topology for the target sequence, even though native-like models exist in the searching space. These proteins in dawn region are low-confidence targets for FR-t5, which included a significant portion of low-homology proteins. In consideration of more conserved features derived directly from a structure model, model evaluation method could provide an avenue to improve the performance of TBM in the dawn region. Here we report a novel model evaluation method called MEFTop that combines traditional features with two groups of newly introduced structural features. The obtained testing results indicate that these novel structural features contribute significantly to the improvement of MEFTop performance in the dawn region. We further show that MEFTop could be combined with FR-t5 and other threading programs to improve the low-homology protein modeling.

## Results

In this section, we will first show the performance improvement of MEFTop for protein targets in the dawn region. Then, we will analyze the contribution of newly introduced structural features in MEFTop. Thirdly, we will explore how the FR-t5-M, the combination of MEFTop with FR-t5, improves modeling for targets in the dawn region and test its performance on CASP10 targets. Finally, we will demonstrate the application of MEFTop to some other threading algorithms such as RaptorX [10] and SPARKS-X [9].

### The Performance of MEFTop in the Dawn Region

**Figure 1:**
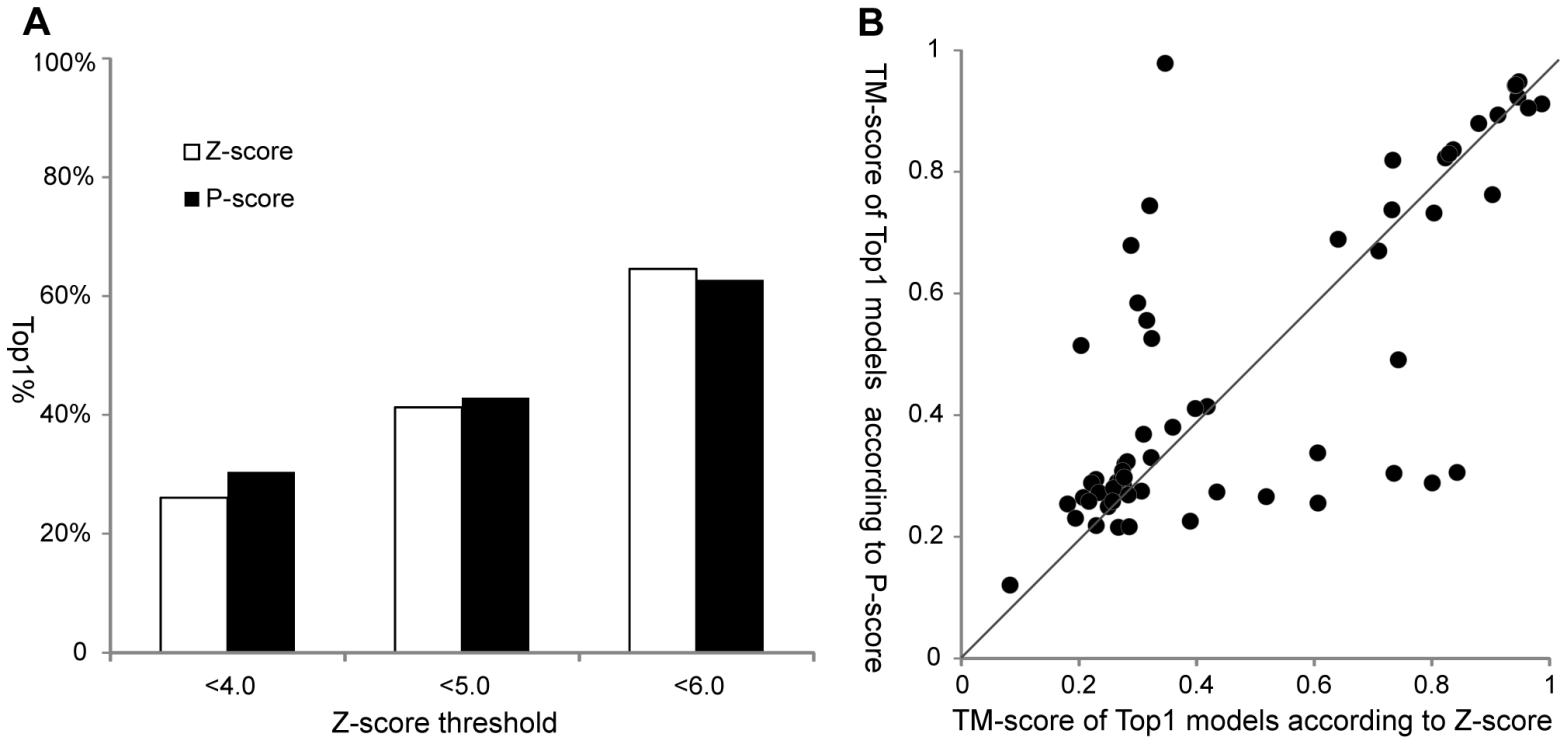
Comparison of the performance of MEFTop and FR-t5 in the dawn region of SCOP1.75–500 set. (A) The percentage of native-like Top1 models (Top1%) that selected by MEFTop using P-score and FR-t5 using Z-score. The X-axis is the Z-score cutoff and the Y-axis is the Top1%. The performances of Z-score and P-score are shown as white and black columns, respectively. (B) The TM-score of Top1 models selected according to Z-score and P-score for 63 targets with optimal Z-score <5.0. The X-axis and Y-axis of each point represent the TM-scores of Top1 models selected by Z-score and P-score, respectively.

To evaluate the robustness of MEFTop, a 5-fold cross-validation was carried out on the training set SCOP1.75-Z6. The average and standard deviation of the percentage of native-like Top1 models (Top1%) (see Methods section for details) was 46.84%±2.55%, which indicates stable performance of MEFTop for targets in the dawn region. The performance of MEFTop was further tested on the data set (SCOP1.75–500) which included 110 proteins in dawn region. The Top1% selected with the P-score of MEFTop was compared to that selected with the Z-score of FR-t5. As shown in Figure 1A, we found that the Top1% selected according to the P-score was higher when the best Z-score cutoff of targets was used as 4.0 or 5.0, but somewhat lower when targets had an optimal Z-score less than 6.0. Furthermore, in order to evaluate the models selected according to P-score and Z-score, we compared the TM-score [25] of Top1 models according to two metrics for targets with an optimal Z-score <5.0 on the SCOP1.75–500 set (Figure 1B). Of 63 targets on the testing set, there were 33 Top1 models with better quality selected according to the P-score, while 22 Top1 models with better quality selected according to Z-score. These results indicate that better performance for protein modeling can be achieved for targets in the dawn region using the P-score of the MEFTop method than using the Z-score of the FR-t5 method.

### The Contribution of Newly Introduced Structural Features in MEFTop

To investigate the contribution of newly introduced structural features of MEFTop to the improvement of model evaluation for dawn region proteins, different combinations of features were trained on the SCOP1.75-Z6 set and tested on the SCOP1.75–500 set. As shown in Table 1, two groups of structural features, including SSE contact features and 3-dimensional topology features, contributed significantly to the improvement of the MEFTop method. When SSE and 3D topology features were added separately, for the targets with an optimal Z-score less than 6.0, the Top1% increased to 56.4% (SSE) and 58.2% (3D topology) as compared to 53.6% when only the traditional features were considered. Similar improvements were also observed for the targets with optimal Z-score less than 4.0 and 5.0. As expected, after incorporating the two groups of structural features with traditional features, the Top1% increased more significantly, from 53.6% to 62.7% for the targets with an optimal Z-score less than 6.0.

**Table 1:** Testing results for the contribution of structural features to MEFTop in the dawn region on SCOP1.75–500 set.

	SCOP1.75–500(Top1%)		
Feature	<6.0*	<5.0*	<4.0*
T	53.6	36.5	23.9
SSE	38.2	27.0	15.2
Topology	43.6	28.6	23.9
T+SSE	56.4	41.2	28.3
T+Topology	58.2	44.4	30.4
SSE+Toplogy	44.5	30.2	23.9
All (T+SSE+Topology)	62.7	42.9	30.4

*Targets with optimal Z-score less than this cutoff value (6.0 or 5.0 or 4.0). On the SCOP1.75–500 set, the numbers of targets are 110 (Z-score<6.0), 63(Z-score<5.0) and 46(Z-score<4.0).

### The Combination of MEFTop with FR-t5, Denoted as FR-t5-M, Significantly Improves the FR-t5 in the Dawn Region

As shown in Figure 1B, although overall the P-score of MEFTop outperforms Z-score of FR-t5 in model selection for the targets in the dawn region, the two metrics apparently showed complementarity. Thus we sought to integrate the two metrics (denoted as M-score) to achieve a better performance of protein prediction by combining the methods MEFTop and FR-t5 (denoted as FR-t5-M) (see Methods section for detailed description).

**Figure 2:**
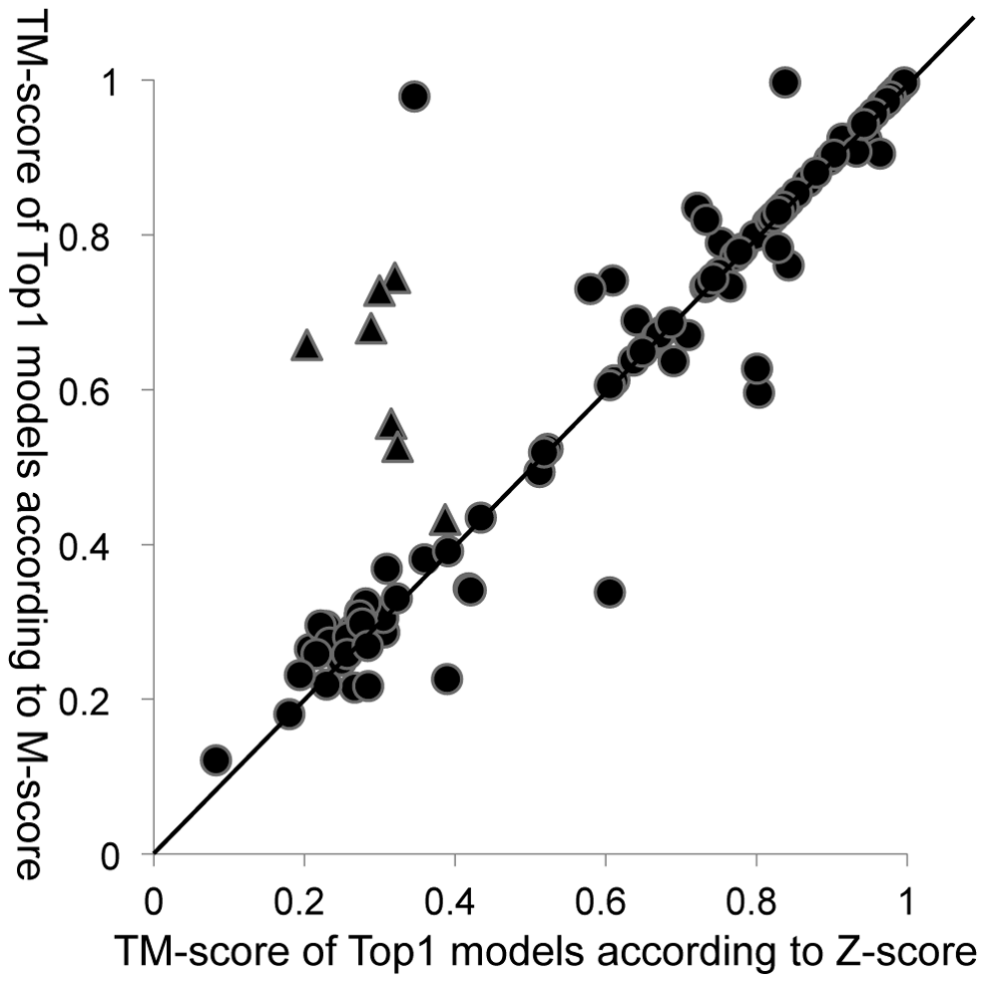
The TM-score of Top1 models selected according to Z-score and M-score for all targets with optimal Z-score <6.0 on SCOP1.75–500 set. The X-axis and Y-axis of each point represent the TM-score of Top1 models selected according to Z-score and M-score, respectively. Low homology proteins (marked by triangles) had high-quality Top1 models by FR-t5-M (M-score) whereas not FR-t5 (Z-score).

**Figure 3:**
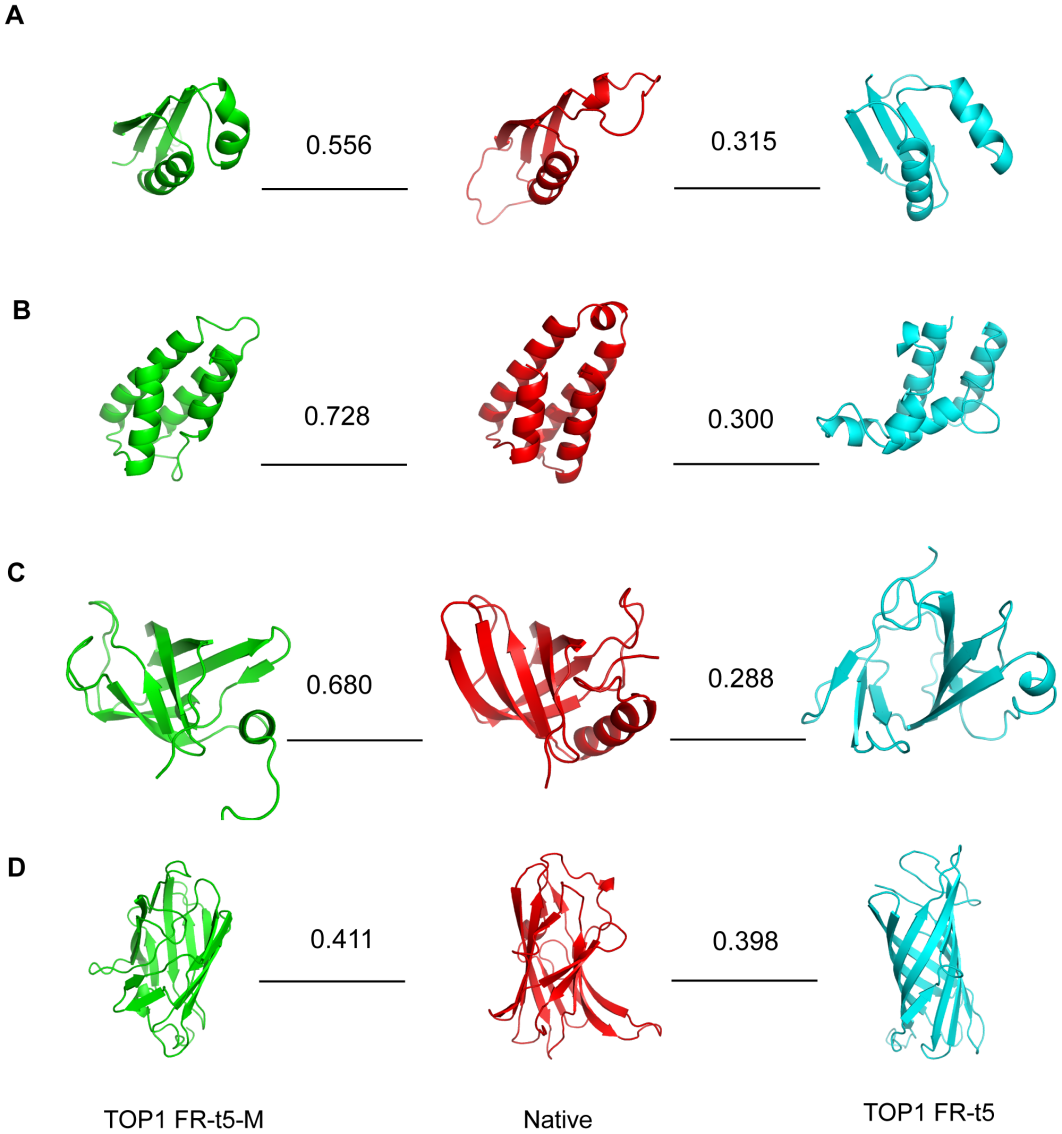
Four representative targets with different Top1 models selected by FR-t5-M (M-score) and FR-t5 (Z-score). The native structure (red) of d1b33n_ (A), d2bl8c1 (B), d2rdeb1 (C) and d1sgka1 (D), the Top1 model selected by FR-t5-M using M-score (green) and FR-t5 using Z-score (cyan) are shown. The TM-scores of Top1 models and native structures are presented. 3D structure models are produced with PyMOL (http://www.pymol.org/).

To evaluate FR-t5-M, we compared the performance of M-score and Z-score for the 110 targets in the dawn region of SCOP1.75–500 (Table 2). From the data presented in Table 2, it is evident that the M-score outperformed the Z-score for all criteria listed. For instance, the average rank of Top1 models (see Methods section for details) was 9.14 for the M-score, whereas it was 11.48 for the Z-score. Figure 2 gives a more detailed comparison of the two methods by looking at the quality of Top1 models according to TM-score. Notably, FR-t5-M could find high-quality models for 7 low homology proteins (marked by triangles), whereas FR-t5 could not. Four of these 7 low homology proteins were illustrated in Figure 3. One example is a bacterial immunity domain d2bl8c1 containing 81 amino acids (AA). The Top1 model selected by FR-t5-M (M-score) has a TM-score of 0.728, which is in higher quality than the model selected by the FR-t5 (Z-score) (TM-score = 0.300). The other three examples are d1b33n_ (67 AA), d2rdeb1 (110 AA) and d1sgka1 (155 AA). Their Top1 models selected by the FR-t5-M (M-score) were all native-like, whereas models selected by the FR-t5 (Z-score) were native-unlike. These differences in model selection between M-score and Z-score revealed that structural features clearly contributed in model evaluation and selection. As shown in Figure 3, all Top1 models selected according to their Z-score also had similar SSEs type to native structures, whereas the topology relationship between these SSEs was not correct. However, the MEFTop algorithm corrected for this error through utilizing the SSE contact map and introducing topological constraints.

**Table 2:** Improvements of FR-t5-M over FR-t5 in the dawn region on SCOP1.75–500 set.

				Top1%		
Metrics	Average^a^	Sum^b^	CC ±σ^c^	<6.0^d^	<5.0^d^	<4.0^d^
Z-score	11.48	66.41	0.472±0.382	64.5	41.3	26.1
M-score	9.14	69.08	0.556±0.344	69.1	50.8	37.0

a The average rank according to TM-score(over 110 decoy sets) in the absence of native structures.

b The sum of TM-scores for Top1 models in the dawn region.

c The average and standard deviation of Pearson correlation coefficients between predicted score and TM-score for every target in the dawn region.

d Targets whose best Z-score is less than the cutoff. On the SCOP1.75–500 set, the number of targets is 110(Z-score<6.0), 63(Z-score<5.0) and 46(Z-score<4.0), respectively.

Since a significant portion of low-homology proteins were included in the dawn regions, we further compared FR-t5-M and FR-t5 on these low-homology proteins. Of 110 proteins in dawn region of SCOP1.75–500, 59 have sequence identity less than 40%. As shown in Table 3, for these 59 targets, the average rank of Top1 models and Top1% were 13.49 and 52.5% for M-score, and 17.72 and 42.4% for Z-score, respectively. The similar improvement was also observed in 25 proteins whose sequence identity less than 30%.

**Table 3:** Improvements of FR-t5-M over FR-t5 for low-homology targets on SCOP1.75–500 set.

	Seq-40%^a^		Seq-30%^a^		
Metrics	Ave-Rank^b^	Top1%^c^	Ave-Rank^b^		Top1%^c^
Z-score	17.72	42.4	17.80	32.0	
M-score	13.49	52.5	16.32	40.0	

a The sequence identity.

b The average rank according to TM-score in the absence of native structures.

c The Top1% is the fraction of native-like Top1 models for all targets.

The FR-t5-M was also evaluated on the 390 targets of high confidence from the SCOP1.75–500 dataset (Table S1). We found that the two methods exhibited similar performances for high-confidence targets.

We further tested the performance of FR-t5-M on targets of the recent CASP10. A comprehensive comparison between the performance of FR-t5-M (M-score) and FR-t5 (Z-score) on the 103 targets of CASP10 data set is shown in Table 4. Overall, FR-t5-M outperformed FR-t5 as measured by average rank (9.00 vs 10.46) and average TM-score (0.570 vs 0.564). Notably, the improvement was contributed by dawn region targets. For the 57 targets in dawn region, the average ranks for FR-t5-M and FR-t5 were 12.08 and 14.15 respectively.

**Table 4:** Performances of FR-t5-M and FR-t5 on CASP10 set.

	All		Dawn region^a^		High-confidence^b^	
Metrics	Ave-Rank^c^	Ave-TM^d^	Ave-Rank^c^	Ave-TM^d^	Ave-Rank^c^	Ave-TM^d^
Z-score	10.46	0.564	14.15	0.449	2.79	0.803
M-score	9.00	0.570	12.08	0.458	2.58	0.802

a 57 proteins whose optimal Z-score <6.0.

b 46 proteins whose optimal Z-score > = 6.0.

c The average rank according to TM-score in the absence of native structures.

d The average of TM-scores for Top1 models.

### The Integration of MEFTop with other Threading Methods

Here we would like to demonstrate that the MEFTop could offer a general approach to improve protein modeling by combining it with another two popular threading programs, RaptorX and SPARKS-X. These two integrated methods RaptorX-M and SPARKS-X-M were tested on the 110 targets in the dawn region of SCOP1.75–500. As shown in Table 5 and 6, both integrated methods were significantly improved. For RaptorX-M, the Top1% increased from 76.0% to 78.8%.

**Table 5:** Improvements of RaptorX-M over RaptorX in the dawn region on SCOP1.75–500 set.

Method	Top1%^a^	Sum^b^	CC ±σ^c^	Average^d^
RaptorX	76.0	69.95	0.572±0.324	13.88
RaptorX-M	78.8	71.4	0.581±0.320	11.81

a The Top1% is the fraction of native-like Top1 models for 104 targets in the dawn region whose optimal Z-score(FR-t5) is less than 6.0. (remove 6 targets which could not get complete models by RaptorX).

b The sum of TM-scores for Top1 models in the dawn region.

c The average and standard deviation of Pearson correlation coefficients between predicted score and TM-score for every target in the dawn region.

d The average rank according to TM-score(over 104 decoy sets, remove 6 targets which could not get complete models by RaptorX) in the absence of native structures.

**Table 6:** Improvements of SPARKS-X-M over SPARKS-X in the dawn region on SCOP1.75–500 set.

Method	Top1%^a^	Sum^b^	CC ±σ^c^	Average^d^
SPARKS-X	70.9	68.64	0.518±0.351	13.05
SPARKS-X -M	73.7	69.93	0.587±0.301	9.77

a The Top1% is the fraction of native-like Top1 models for 110 targets in the dawn region whose optimal Z-score(FR-t5) is less than 6.0.

b The sum of TM-scores for Top1 models in the dawn region.

c The average and standard deviation of Pearson correlation coefficients between predicted score and TM-score for every target in the dawn region.

d The average rank according to TM-score(over 110 decoy sets) in the absence of native structures.

We further looked into the performance of the newly integrated methods (RaptorX-M and SPARKSX-M) on 59 low-homology targets (sequence identity less than 40%) in SCOP1.75–500. From the data presented in Table 7, for RaptorX-M, the Top1% increased from 63.2% to 66.7%.

**Table 7:** Improvements of RaptorX-M and SPARKS-X-M for low-homology targets on SCOP1.75–500 set.

	Seq-40%^a^		Seq-30%^a^	
Method	Top1%^b^	Ave-Rank^c^	Top1%^b^	Ave-Rank^c^
RaptorX	63.2	20.53	54.2	25.38
RaptorX-M	66.7	17.07	62.5	19.38
SPARKS-X	47.5	15.31	32.0	20.64
SPARKS-X-M	49.2	10.85	40.0	11.52

a The sequence identity.

b The Top1% is the fraction of native-like Top1 models for all targets.

c The average rank according to TM-score in the absence of native structures.

*Note:* Ave-Rank is only compared between a pair of methods (RaptorX/RaptorX-M and SPARKS-X/SPARKS-X-M).

## Discussion

In order to improve low-homology protein modeling, we have developed a useful model evaluation method (MEFTop) by focusing on evaluating the native-likeness of topology. Further, by incorporating MEFTop with our previously developed threading method FR-t5, a new TBM method (FR-t5-M) was developed. We found that FR-t5-M significantly outperforms our previous threading method FR-t5, and displays a predictive performance for low-homology CASP10 targets that is comparable to most other popular protein structure prediction programs. Moreover, we observed significant improvements in predicting structures for low-homology proteins when combining the MEFTop with RaptorX and SPARKS-X. Taken together, we argue that MEFTop could offer a generalized method to improve threading algorithms for low-homology protein modeling.

A wide range of earlier studies have demonstrated that traditional features of 1D and 2D information can be effectively utilized for high-quality model evaluation [15], [19], [21]. Our research revealed that the integration of SSE contact features and 3D topology features into the model evaluation method MEFTop greatly increased the quality of model evaluation for proteins in the so-called dawn region. The incorporation of these two groups of structural features was intended to capture the topology structure information during evaluation of the quality of models. As shown above, the introduction of these structural features significantly improves the percentage of native-like Top1 models in the dawn region or for low homology proteins.

Whilst we have shown that the application of MEFTop or FR-t5-M brings significant improvements, both methods can be optimized further. First, models of FR-t5-M could be optimized with the introduction of side-chain packing and refinement in the future. Second, a systematic and complete programming code optimization should result in accelerating the program. As a case in point, a mutation in the transporter membrane protein SLC45A2, which is the genetic basis of the fur color of white tigers, was successfully predicted by using FR-t5-M [26]. In summary, both our model evaluation method MEFTop and improved TBM program FR-t5-M could facilitate a wide range of applications.

## Materials and Methods

### Data Set

The CASP7-8 data set was used as training data, which consists of 221 CASP7 and CASP8 targets (http://predictioncenter.org/). The CASP10 data set was used as testing data, which includes 103 targets in total (http://predictioncenter.org/).

For further training and testing, another two datasets, the SCOP1.75-Z6 as training data and SCOP1.75–500 as testing data were constructed from SCOP1.75 [27], independently. The SCOP1.75-Z6 set was constructed as follows. Firstly, 1401 domains over 1195 fold classes were selected uniformly as the size of fold class. Then, 252 targets in the dawn region (optimal Z-score <6.0 for FR-t5) were kept. Similarly, the SCOP1.75–500 set consists of 500 domains covering 307 folds was built. Notably, a major difference between the two data sets is that the SCOP1.75-Z6 set only includes targets in the dawn region, while the SCOP1.75–500 set is a comprehensive set that consists of high-confidence targets, as well as targets in the dawn region. The SCOP1.75-Z6 and SCOP1.75–500 data set were available at http://jianglab.ibp.ac.cn/lims/MEFTop/meftop.html.

For each protein in training and testing data, 50 structural models were generated by FR-t5.

### Feature Extraction and SVM Predictor

MEFTop was developed as an SVM predictor that considered 37 features classified into four groups: (1) 1-dimensional (1D) and (2) 2-dimentional (2D) contact map features, (3) Secondary Structural Element (SSE) contact features and (4) 3-dimensional topology features.

1D features included secondary structure (SS) represented by helix, strand and coil and relative solvent accessibility (RSA) computed as exposed and buried states. For a target sequence, its SS state and RSA state for each residue were predicted by SCRATH [28]. For each structural model of the target sequence, the SS state and RSA state were calculated for each residue with DSSP [29]. Then the percentages of residues of the three SS states (helix%, strand% and coil%) and of the two RSA states (exposed%, buried%) were calculated over all the residues for both target sequence and its structural models. Thus we obtained 10 1D features for both sequence and structural models. Based on these 1D features, four similarity scores between the target sequence and its structural model were derived by following Wang and colleagues’ work [21]. More specially, the 1D features (the percentages of helix, strand, coil, exposed and buried) of target sequence and its structural model can be regarded as two composition vectors. The cosine, correlation, Gaussian kernel, and dot products of the two composition vectors were calculated as four similarity scores, namely 4 features. In total, there were 14 features derived as 1D features.

2D contact map features capture contact information between residues with separation ≥6 residues at two distance thresholds (<8Å and <12Å) between the side chain center of mass (SCM) [21]. For a target sequence, the contact probability of each residue pair was predicted by SCRATH, while the information about a residue pair in contact or not was readily extracted from structural models. Then for each residue in target sequence or structural models, its contact order and contact number were calculated as 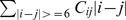 and 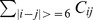 respectively (*C_ij_* is the predicted contact probability from target sequence or extract contact information from structural models for residues *i* and *j*). Thus, the residues contact order of target sequence and its structural model can be regarded as two composition vectors. The cosine and correlation of the two composition vectors were calculated as two similarity scores at a distance threshold. Similarly, another 2 similarity scores were obtained for contact number.

In addition, the overall match score (*f*_res_) of the contact probability between target sequence and structural model was calculated as the following equation:
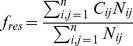
(1)


Here, *n* is the length of sequence. For residues *i* and *j*, *C_ij_* is the predicted contact probability and *N*_ij_ is the contact value from structural model (1 is in contact and 0 is in isolation).

Therefore, to describe the extent of correspondence between a target sequence and its structural model, ten features including eight similarity scores for contact order and contact number and two overall match scores were derived at two distance thresholds. In total, 24 traditional 1D and 2D features were generated.

**Figure 4:**
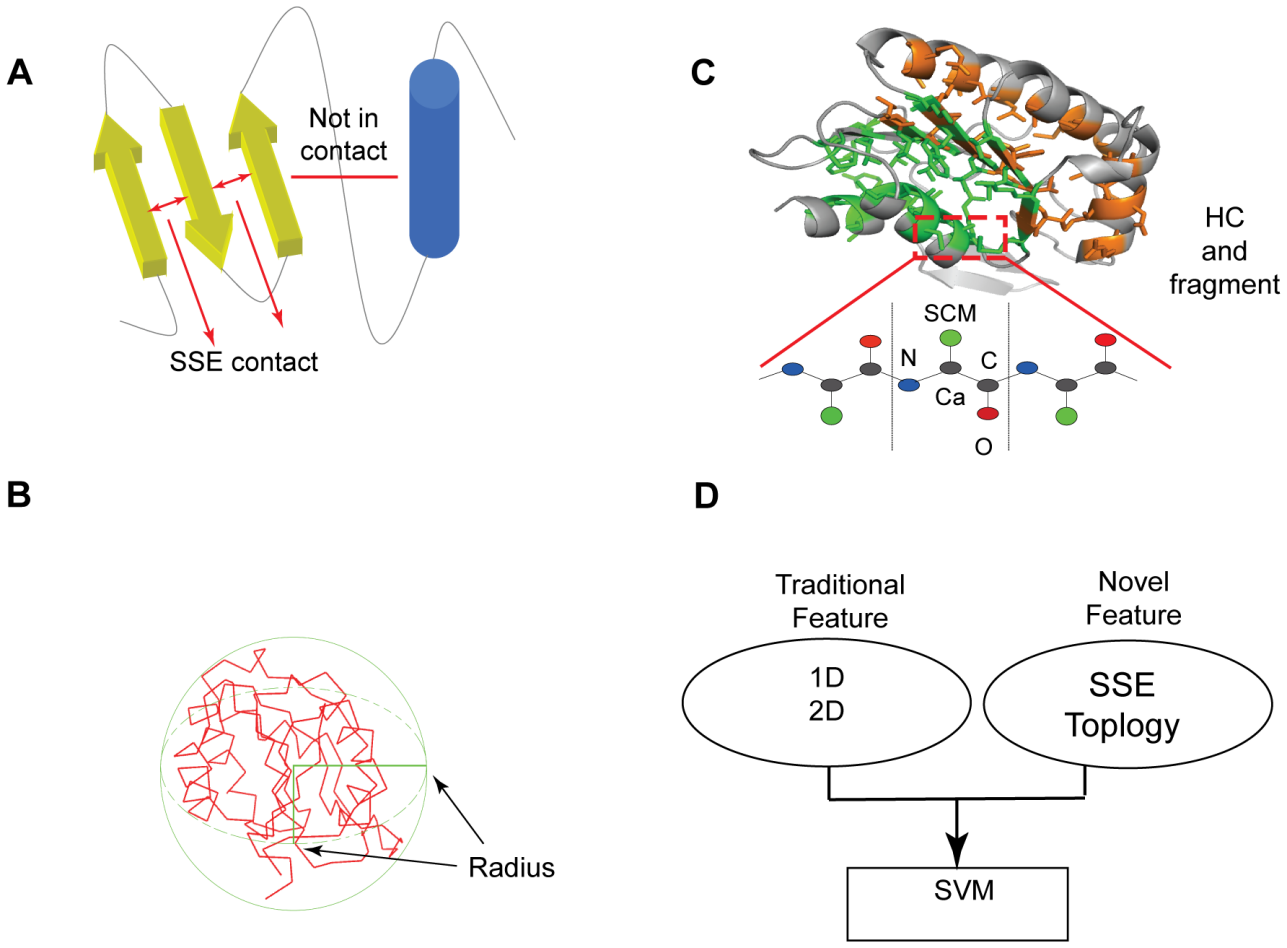
The overview of MEFTop. (A) The cartoon representation of two contacts between two pairs of SSEs (beta strands). (B) The radius of gyration for the model structure as one of the topology features. (C) Hydrophobic core and local conformation potential based on residue fragments. Schematic representation of the backbone atoms (N CA C O) and the side chain center of mass is shown. (D) The SVM predictor. Four groups of input features: traditional sequence (1D) and contact map (2D) features and two groups of newly introduced structural features including SSE contact features and topology features.

SSE contact features capture the information of SSE spatial relationship including the SSE pairs in contact, the distances between SSEs and the SSE lengths. Based on the SS states of residues calculated above, an SSE was identified as a segment consisting of at least 4 continuous residues with helix or strand state. Figure 4A illustrated the cartoon representation of two contacts between two pairs of beta strands. For structural models, the contact strength of two SSEs was computed as the number of residues pairs in contact (distance threshold <8.5Å). For a target sequence, the contact strength of two SSEs was computed as the sum of their residues contact probability (threshold <8Å). An SSE of a structural model was considered to be corresponding to an SSE of the target sequence, if the two SSEs have minimum difference in the starting residue position according to the sequence order. Only the SSEs that have correspondence in both structural model and target sequence were considered in the following calculations. Then, the overall match score (*f*_SSE_) of the SSE contact strength between structural model and target sequence was calculated in the following equation:
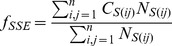
(2)


Here, *n* is the total number of corresponding SSEs between a structural model and target sequence. *C_S(ij)_* is the predicted contact strength of SSE *i* and *j* from target sequence divided by the length of SSE *i*, and *N_S(ij)_* is the contact strength between SSE *i* and *j* extracted from structural model divided by the length of SSE *i*.

Two composition vectors of SSE contact numbers were generated, respectively, for the structural model *F_M_* = [*P_M(1)_*, *P_M(2)_*,…, *P_M(n)_*] (
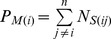
 is the sum of contact strength for SSE *i* in the structural model ) and the target sequence *F_T_* = [*P_T(1)_*, *P_T(2)_*,…, *P_T(n)_*] (
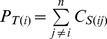
is the sum of contact strength for SSE *i* in the target sequence) and then transformed into similarity scores using the cosine and correlation function.

The overall match of the distances of SSE pairs between the structural model and target sequence was also considered. First, the distance of an SSE pair in structural model was assigned with the minimum distance between residues of this SSE pair, and the distance of SSE pair for the target sequence was estimated from its residue predicted contact probability as follows:
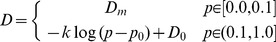
(3)


Here, *D* is the predicted distance of a SSE pair, *p* is the maximum predicted contact probability between the residues of a SSE pair, *D_m_* is the distance threshold, *k* is a constant, and *P_0_* and *D_0_* denote ideal status values. Then, the similarity score of SSE pair distance between the structural model and target sequence was calculated as by following equation S3 (see Methods S1).

The length of the corresponding SSEs between the structural model and target sequence was compared and transformed into two different ratios by equation S6 and S7 (see Methods S1). As seen from above, 6 SSE contact features were generated, including one overall match score (*f*_SSE_) of the SSE contact strength, two similarity scores for SSE contact numbers, one similarity score of SSE pair distance, and two different ratios of SSE lengths.

As shown in Figure 4B and 4C, the topology features were generated from radius of gyration, Hydrophobic Core (HC) and local conformation potential of all fragments for a structure model. To capture the topology compactness, the radius of gyration for each structural model was calculated (Figure 4B). On the other hand, the radius of gyration could be predicted based on the length of the target sequence according to the following equation [30]:
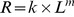
(4)


Here, *R* is the predicted radius of gyration, *L* is the length of sequence, *k* and *m* are constant parameters. The radii of gyration predicted from the target sequence and extracted from the structural model were compared and transformed into two similarity scores by equation S8 and S9 (see Methods S1).

Besides radius of gyration constraints, some local interactions played important roles in protein folding and topology stability, such as hydrophobic interaction. Thus, specific local hydrophobic residue clusters were defined as Hydrophobic Core (HC), and the HC is a new structural descriptor (Figure 4C). The radius, the number of hydrophobic residues and the number of SSEs in HC were compared to those in structural model, and transformed into three 3D topology features. In addition, potentials from the local conformation of fragments (Figure 4C) [31] were also used as 2 features. In total, 7 features were obtained for describing the 3-dimensional topology.

Figure 4D illustrates the use of SVM predictor as a core component of MEFTop to evaluate model quality. The SVM predictor takes as inputs the traditional 1D and 2D residue contact map features and two groups of additional structural features. Thus MEFTop represents a novel model evaluation and selection program with focus on predicting the similarity in topology between a predicted model and its native structure.

### Evaluation Score

In the FR-t5 program, the Z-score was applied for template ranking, and could also be used to assist in the selection of the optimal structural model. The raw score 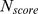
of the FR-t5 scoring function [7], which is positively correlated with the quality of the alignment between query and template sequences, was transformed into a Z-score as follows:
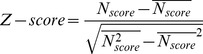
(5)


Here 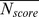 is the average of 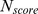, and 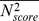 is the mean square of 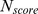.

In MEFTop, a P-score was used to evaluate the quality of a structural model through a SVM regression function 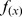 as follows:
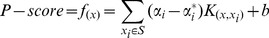
(6)


Here, the value computed by 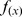 is the estimate of the TM-score associated with an input feature vector 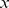 of a model. 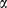 and 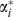 are non-negative weights assigned to the training data point 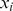, and they control the trade-off between training errors and the smoothness of f(x) during training [32]. b represents the bias term. K is the kernel function, which could be viewed as a function to compute the similarity between the training data point 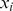 and the target data point 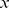. The function related parameters were optimized on the training set.

In order to form the new modeling program FR-t5-M, MEFTop was combined with FR-t5. A new metric called *M-score* was then used as follows:
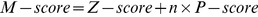
(7)


Here, *n* is the weight for *P-score*.

### Training and Testing

MEFTop was firstly trained and evaluated as a general model evaluation method in our research. The training dataset was CASP7-8 set, which is generated from 221 targets of CASP7 and CASP8 with FR-t5. Furthermore, in order to adapt this method for targets in the dawn region, MEFTop was optimized using the SCOP1.75-Z6 set. First, the weight for the vector of this structural model was assigned according to its TM-score, and the weights and features were used as inputs for the software LIBSVM [33]. Basically, a bigger TM-score represents a larger weight. Subsequently, the SVM predictor was trained and optimized with a cost function (*F*) as follows:
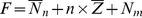
(8)


Here, 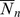 is the average rank of native structure, 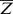 is the average Z-score in SVM (*Z-score* ^SVM^) for training target, *n* is the weight of *Z-score* ^SVM^ and 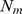 is number of missed proteins whose native structures have not been ranked 1st. The optimization goal was to minimize the cost function value. To evaluate the robustness of the SVM predictor, a 5-fold test for the dataset SCOP1.75-Z6 was carried out.

After training of MEFTop using the above process, the performance of MEFTop was tested on the SCOP1.75–500, with particular focus on targets in the dawn region. Mostly, two criteria were used to evaluate the performance of evaluation method. They include the percentage of native-like Top1 models (Top1%) and the average rank of Top1 models. The Top1% is the fraction of native-like Top1 models for all targets. If the TM-score of a model is larger than 0.4, the model is usually defined as a native-like model, which has a similar topology when compared with its native structure [34]. The average rank represents the average value for the rank of the selected model in all potential models for a target, according to its TM-score.

Similar to MEFTop, our new method FR-t5-M (using M-score as a metric) was optimized on the SCOP1.75-Z6 dataset, and evaluated on both the SCOP1.75–500 and CASP10 datasets.

### Evaluation of the Combination of MEFTop with RaptorX and SPARKS-X

Similar to the Z-score of FR-t5, a score of RaptorX and an energy score of SPARKS-X were used to rank templates, respectively. We integrated the P-score of MEFTop with the rank score of these threading programs into new metrics similar to the M-score of FR-t5-M. Then these new methods which combined MEFTop with threading programs (RaptorX and SPARKS-X) were evaluated for 110 targets in the dawn region of SCOP1.75–500. Among the 110 targets, 59 targets have sequence identity to templates less than 40%, and 25 targets less than 30%. For each protein in training and testing data, 100 structural models were generated by RaptorX and 80 structural models were generated by SPARKS-X.

## Supporting Information

Table S1 Performances of FR-t5-M and FR-t5 on targets of high confidence from the SCOP1.75–500 dataset.
(DOC)

Methods S1 Equations for Feature Extraction.
(DOC)
