# Supplementary material for: Improvement in Low-Homology Template-Based Modeling by Employing a Model Evaluation Method with Focus on Topology
Source: PLoS One. 2014 Feb 26;9(2):e89935. doi: 10.1371/journal.pone.0089935 (PMC3935967; doi:10.1371/journal.pone.0089935)
Supplement: Table S1 — Performances of FR-t5-M and FR-t5 on targets of high confidence from the SCOP1.75–500 dataset. (DOC) [file pone.0089935.s001.doc]

**Table S1.** Performances of FR-t5-M and FR-t5 on targets of high confidence from the SCOP1.75-500 dataset.

| Metrics |  | Average^a^ | Sum^b^ |
| --- | --- | --- | --- |
| Z-score |  | 1.49 | 357.54 |
| M-score |  | 1.49 | 357.54 |

a The average rank according to TM-score in the absence of native structures for the whole set or high region.

b The sum of TM-scores for Top1 models on the SCOP1.75-500 set or high region.

*Note:* 390 high confidence targets whose best Z-score >=6.0.
