# Supplementary material for: Improvement in Low-Homology Template-Based Modeling by Employing a Model Evaluation Method with Focus on Topology
Source: PLoS One. 2014 Feb 26;9(2):e89935. doi: 10.1371/journal.pone.0089935 (PMC3935967; doi:10.1371/journal.pone.0089935)
Supplement: Methods S1 — Equations for Feature Extraction. (DOC) [file pone.0089935.s002.doc]

Supporting Methods S1

Equation for Feature Extraction

Equation S1, S2 andS3

$qs_{(i,j)}=\exp\left\{-\left[\frac{D_{Q(i,j)}-D_{M(i,j)}}{D_{Q(i,j)}}\right]^{2}\times\ln2\right\}$ (S1)

$ms_{(i,j)}=\exp\left\{-\left[\frac{D_{Q(i,j)}-D_{M(i,j)}}{D_{M(i,j)}}\right]^{2}\times\ln2\right\}$ (S2)

$S_{D}=\frac{\sum_{i,j} P_{Q(i,j)}qs_{(i,j)}+\sum_{i,j} P_{M(i,j)}ms_{(i,j)}}{\sum_{i,j} P_{Q(i,j)}+\sum_{i,j} P_{M(i,j)}}$ (S3)

In the above equation, *D_M(i,j)_* and *D_Q(i,j)_* were the distance of SSE pair (*i* and *j*) for the structural model and target sequence, respectively. *P_M(i,j)_* and *P_Q(i,j)_* were the contact strength between SSE *i* and *j* for the structural model and target sequence, respectively. S_D_ in equation S3 is the similarity score used as input feature.

Equation S4, S5, S6 and S7

$S_{len(i)}=\exp\left\{\left[\frac{L_{Q(i)}-L_{M(i)}}{0.25*L_{Q(i)}}\right]^{2}\times\ln2\right\}$ (S4)

$S_{lt(i)}=\left\{\begin{matrix} 0.5\times S_{len(i)} & (Q(i),M(i))\in(H,E)or(E,H) \\ S_{len(i)} & (Q(i),M(i))\in(H,H)or(E,E) \end{matrix}\right.$ (S5)

$S_{L1}=\frac{\sum_{i=1}^{n} L_{M(i)}S_{len(i)}}{\sum_{i=1}^{n} L_{M(i)}}$ (S6)

$S_{L2}=\frac{\sum_{i=1}^{n} L_{M(i)}S_{lt(i)}}{\sum_{i=1}^{n} L_{M(i)}}$ (S7)

In the equation S4-S7, *L_M(i)_* and *L_Q(i)_* were the length of SSE *i* for structural model and target sequence, respectively. *n* is the number of corresponding SSEs in the protein. One similarity score is calculated as *S_L1_* in equation S6, and the other is considered different weights for helix and strand as *S_L2_* in equation S7.

Equation S8 and S9

$S_{r1}=\exp\left\{-\left[\frac{R_{M}-R_{Q}}{0.5\times(R_{M}+R_{Q})}\right]^{2}\times\ln2\right\}$ (S8)

$S_{r2}=\exp\left\{-\left[\frac{R_{M}-R_{Q}}{0.5\times R_{Q}}\right]^{2}\times\ln2\right\}$ (S9)

In the above equation, *R_Q_* is the predicted radius of gyration from target sequence and *R_M_* is the radius of gyration extracted from the structural model. One similarity score is normalized by the sum of radius of the structural model and sequence as *S_r1_* in equation S8, and the other score is only normalized by the predicted radius of the sequence as *S_r2_* in equation S9.
